# The Slaughter of Teeth

**Published:** 1920-04

**Authors:** 


### THE SLAUGHTER OF TEETH

Slaughter implies ruthlessness, wantonness, unnecessary destruction, and apparently the term is correctly descriptive of what has happened. Voices are being raised against the procedure and those who practice it—voices that are not as welcome as they might be because they are voices from the outside. We recall that before external criticisms have been made against the dental profession, and we also recall our resentment when they occurred. Again the faultfinding voice is from the medical profession. "The whole trouble seems to be the lack of good judgment on the part of dentists who are going to the extremes in not desiring to take the consequences of leaving focal infection in the mouth." A dentist whose name is familiar in our journals voices the following: "If the dental profession as a whole does not cease handling pulpless teeth in the incompetent manner in which we know from an examination of thousands of cases they have been handled, then the medical profession will exercise their prerogative and say when we shall and when we shall not handle pulpless teeth." Imagine such a thing!

Since the findings of Rosenow and Billings, with the showings of the radiograph to encourage us, we have extracted with high resolve. To add to our enthusiasm, we have had reports from all sides of very remarkable cures of various baffling ills as a result of our extractions. Certainly a tooth should get no consideration when the general health was jeopardized! And for the most part it got none. No count has been officially reported of the number of times teeth have been removed with the best of motives and following quite far-reaching promises in some instances with no results at all. Still we have not been startled into conservation, although putting a tooth back after extraction has disappointed us and has never become popular. Perhaps our confidence in artificial restoratives has comforted us.

And to leave a bad matter still bad, we have no authority whose word can be final in this matter. Orthodoxy in science is not yet. Considering suppurative pericementitis and apical abscesses as the principle causes of extraction, dental opinion as voiced by those whose voices are heard the oftenest, and by that token thought by many to be authoritative, is a quagmire of contradiction, misinformation, unsupported opinion and pseudo-science. To get a conception of our status on this most important subject, one needs only to read the current offerings of our magazines.

And furthermore medicine has helped us. Quick to accept the findings of the leaders in bacteriological and pathological research the great parent profession was as quick to put the findings to effect, with the result that daily patients were sent to the radiographer and thence to the dentist with orders for extraction. It was a good beginning; the beginning was made where it was the easiest and the patient was duly impressed with the up-to-date intelligence of the physician. There was no palliative consideration for the teeth, for what is a mere tooth? And the dentist, desiring to be no whit behind the physician, extracted. In this combination the dentist was to blame. He is supposed to know something about teeth, while the physician knows nothing about them. What the schools give medical students about dental tissues and diseases is a travesty.

Another collaborator in the slaughter was the radiogram—the elusive shadow with its enigmatic suggestion of ill-aided and abetted edentulousness. For a long enough period the film and forceps might have been the dental coat-of-arms. And who would hang his honor to a film to-day. But the skiagram is the last word in science and we are scientists, so—exeunt. Often the radiographer returned a diagnosis with the film until dental pride grew tender, and then with ethical religiosity he suggested "diagnosis upon request." And frequently the X-ray man was not a dentist!

All the while there have been conservatives who urged that we were too free to extract. There are men in our profssion whose words are always "apples of gold" if we would only listen to them. Thumbing our literature it is easy enough to find them, words of council, words of fear—fear of the far swing of the pendulum. In their long experiences they have seen great enthusiasms sweep over the profession before, only to leave a wake of shattered hopes and a clientele rich in resentment. They foresaw this day, too, and urged wisdom and discretion, only to be regarded too often as old-fashioned and behind the times. Also these men do not run to print as do so many whose flow of new thought effects a screen for hiding previous ignorance.

Dentists must realize that no substitute they can make can take the place of the teeth they extract. They must learn that the patient sent by the physician for extractions has other possible sources of infection besides the mouth, and that mouth sanitation might be attempted to show whether the mouth is the contributing cause or whether there is some other. "The individual," says one, "who advocates complete destruction of the dental organs in order to rid the body of streptococcal growths evidences profound ignorance." And he might have added that the dentist who extracts without giving the case the most complete examination before extracting, except in the obvious instance, commits a crime. Heart disease, frequently caused by oral infection, is a greater menace than tuberculosis, but that does not justify extraction without a little diagnostic cerebration.—Editorial in March *Journal of the National Dental Association.*

